# Runge-Kutta 4^th^-order method analysis for viscoelastic Oldroyd 8-constant fluid used as coating material for wire with temperature dependent viscosity

**DOI:** 10.1038/s41598-018-32068-z

**Published:** 2018-09-28

**Authors:** Zeeshan Khan, Haroon Ur Rasheed, Iskander Tlili, Ilyas Khan, Tariq Abbas

**Affiliations:** 1 Faculty of Mathematics and Statistics, Ton Duc Thang University, Ho Chi Minh City, Vietnam; 2 Sarhad University of Science and Information Technology, 25000 Peshawar, KPK Pakistan; 3 Energy and Thermal Systems Laboratory, National Engineering School of Monastir, Street Ibn El Jazzar, 5019 Monastir, Tunisia

## Abstract

Polymer flow during wire coating dragged from a bath of viscoelastic incompreesible and laminar fluid inside pressure type die is carried out numerically. In wire coating the flow depends on the velcocity of the wire, geometry of the die and viscosity of the fluid. The governing equations expressing the heat transfer and flow solved numerically by Runge-Kutta fourth order method with shooting technique. Reynolds model and Vogel’s models are encountered for temperature dependent viscosity. The umerical solutions are obtained for velocity field and temperature distribution. It is seen that the non-Newtonian parameter of the fluid accelerates the velcoty profile in the absence of porous and magnetic parameters. For large value of magnetic parameter the reverse effect is observed. It is observed that the temperature profiles decreases with increasing psedoplastic parameter in the presence and absence of porous matrix as well as magnetic parameter. The Brinkman number contributes to increase the temperature for both Reynolds and Vogel’smmodels. With the increasing of pressure gradient parameter of both Reynolds and Vogel’s models, the velocity and temperature profile increases significantly in the presence of non-Newtonian parameter. The solutions are computed for different physical parameters. Furthermore, the present result is also compared with published results as a particular case.

## Introduction

The understanding and analysis of non-Newtonian fluids is of excellent interest from fundamental as well as applied perspective^1,2^. The knowledge of physics and material science associated with the flows of non-Newtonian fluids may have direct effects on numerous fields such as polymer preparation, covering and coating, ink-jet printing, smaller scale fluidics, homodynamic, the flow of turbulent shear, colloidal and additive suspensions, animal blood etc. For this reason, concentration has been shown towards these flows and subsequently the literature presents extensive work on numerical, analytical, and asymptotic solutions on the subject^3,4^. Furthermore, flow of these fluids presents great challenges equally to the experts from diverse research fields such as numerical simulations, engineering, mathematics, physics etc. Indeed, the equations proposed and created for non-Newtonian models are considerably more complicated as compared with Newtonian fluids. The modeled equations of non-Newtonian fluid are highly nonlinear and very difficult to obtain the exact solutions of such equations^5–8^. Due to the non-linear and inapplicable nature in terms of superposition principle, it is evenly hard to obtain the highly accurate solutions for viscous fluids^5–8^. For this purpose, many researchers developed analytical and numerical technique to obtained the solution of these non-linear equations.

Wire-coating (an extrusion procedure) is generally utilized as a part of the polymer industry for insulation and it protects the wire from mechanical damage. In this procedure an exposed preheated fiber or wire is dipped and dragged through the melted polymer. This procedure can also be accomplished by extruding the melted polymer over a moving wire. A typical wire coating equipment is composed of five distinct units: pay-off tool, wire pre-heating tool, an extruder, a cooling and a take-off tool. Most common dies used for coatings are: tubing-type dies and pressure type dies. The later one is normally used for wire-coating and seems like annulus. That’s why flows through such die are similar to the flows through annular area formed by a couple of coaxial cylinders. One of the two cylinders (inner cylinder) moves in the direction of axial while the second (external cylinder) is fixed. Preliminary efforts done by several researchers^9–14^ used power-law and Newtonian models to reveal the rheology of the polymer melt flow. In^15,16^, authors provide the wire coating analysis using pressure type die. Afterwards, more research work regarding this matter was conveyed by^17–20^. In addition, a very detailed survey regarding heat transfer and fluid flow in wire-coating is given by^21^. Akter and Hashmi in^22^, analyzed the wire coating using pressurized die. Later on, Akter and Hashmi performed simulations on polymer flow during the wire-coating process by means of a conical unit as in^23,24^.

Wire coating or covering is a modern industrial procedure to coat a wire for insulation and environmental safety. Wire coating can be classified in three types such as dipping process, coaxial process, and electrostatic deposition process. The dipping process provides considerably stronger bond among the continuums however this process is relatively slow when compared to the other two processes. The problems related to the coating extrusion based on the pressure-die were reviewed by Han and Rao in^25^. Generally, the extrusion process is consisting of three components termed: feeding unit, the barrel and head with a die. The detailed discussions regarding these three distinct elements are reported by Kozan in^26^. In addition, Sajid *et al*. in^27^ addressed and solved the wire-coating process of Oldroyd (8-constant fluid) by utilizing HAM method. Similarly, another research work, presented in^28^ by Shah *et al*. utilized the perturbation approach and analytically analyzed the wire-coating of a third-grade fluid.

At present, the Phan-Thein-Tranner (PTT) model, a third-grade visco-elastic fluid model, is the most commonly used model for wire-coating. The high-speed wire-coating process for polymer melts in elastic constitutive model was analyzed by Binding in^29^. It also discussed the shortcomings of the realistic modeling approach. Multu *et al*. in^30^ provided the wire-coating analysis based on the tube-tooling die. Kasajima and Ito in^31^ analyzed the wire-coating process and examined the post treatment of polymer extruded meanwhile. They also discussed the impacts of heat transfer on cooling coating. Afterward, Winter in^32,33^ investigated the thermal effect on die both from inside and outside perspective. Recently, wire-coating in view of linear variations of temperature in the post-treatment analysis was investigated by Baag and Mishra in^34^.

The wet-on-dry(WOD) and wet-on-wet (WOW) are the most common coating process used for double-layer coating. In wet-on-wet process the fiber is dragged into the primary layer and then curved by ultraviolet lamp. Then, primary coated layer is passed through the secondary coating-layer and again curved by ultraviolet lamp. But in the wet-on-wet process the fiber is passed from the primary and secondary-coating die and then curved by ultraviolet lamp. Recently WOW process gained significant importance production industry. Herein, WOW coating-process is applied for fiber optics. Kim *et al*.^35^ used WOW process for the analysis of two-layers coating on optical fiber. Zeeshan *et al*.^36,37^ used pressure coating die for the two-layer coating in optical coating analysis using PTT fluid model. The same author discussed viscoelastic fluid for two-layer coating in fiber coating^38^. Sisko fluid model was used for fiber coating by adopting WOW process by Zeeshan *et al*.^39^ in the presence of pressure type coating die.

In the recent study MHD flow of viscoelastic fluid is investigated for two-layer coating on optical fiber by applying WOW coating-process inside pressurized coating die. The modeled equations describing the polymer flow inside the die are solved numerically by Runge-Kutta Fehlberg algorithm with shooting technique^40–44^. At the end for validation of the present result, a comparison is done with published results reported by Shah *et al*.^45^.

Sheikholeslami and Ganji^46^ carried out heat transfer enhancement in an air to water heat exchanger with discontinuous helical turbulators; experimental and numerical studies. Sheikholeslami and Ganji^47^ studied heat transfer improvement in a double pipe heat exchanger by means of perforated turbulators. Sheikholeslami *et al*.^48^ investigated heat transfer improvement and pressure drop during condensation of refrigerant-based nanofluid; an experimental procedure. Sheikholeslami and Rokni^49^ investigated CVFEM for effect of Lorentz forces on nanofluid flow in a porous complex shaped enclosure by means of non-equilibrium model. Chahtour *et al*.^50^ studied convective hydromagnetic instabilities of a power-law liquid saturating a porous medium: Flux conditions. Sheikholeslami and Shehzad^51^ carried out Simulation of water based nanofluid convective flow inside a porous enclosure via non-equilibrium model. Sheikholeslami and Seyednezhad^52^ investigated simulation of nanofluid flow and natural convection in a porous media under the influence of electric field using CVFEM.

## Modeling of the Problem

**Figure 1:**
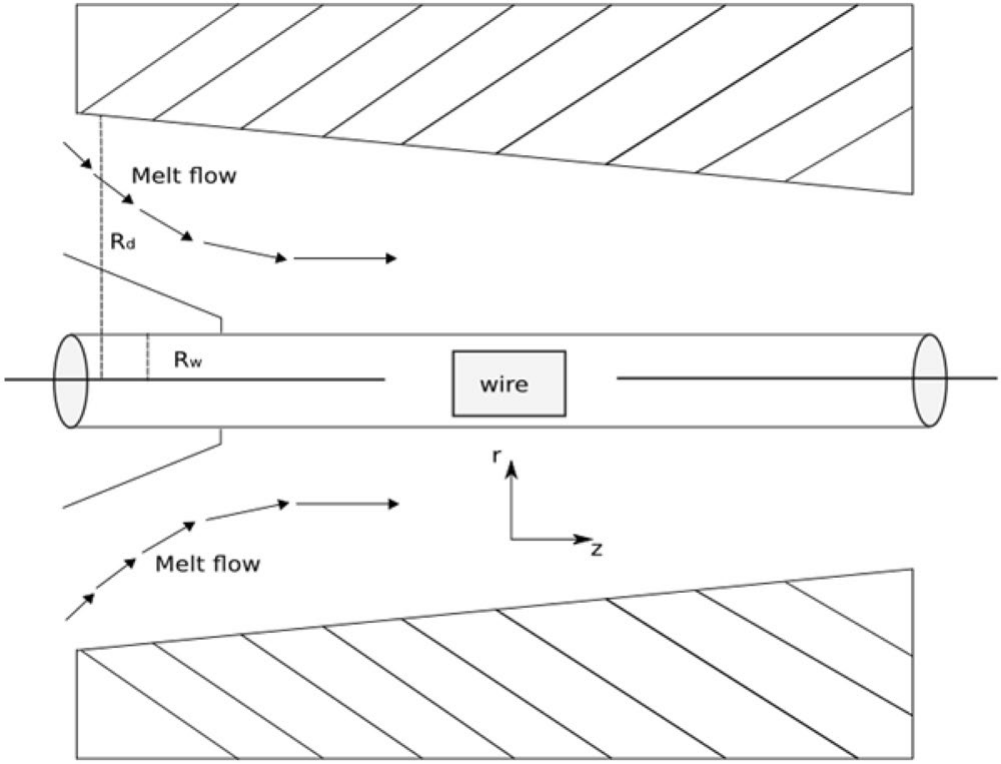
Geometry of wire coating process.

The geometry of the flow problem is shown in Fig. 1 in which the wire of radius *R*_*w*_ is dragged with velocity *V* inside the coating die filled with viscoelastic fluid. The fluid is electrically conducted in the presence of applied magnetic field B_0_ normal to the flow. Due to small magnetic Reynold number the induced magnetic field is negligible, which is also a valid assumption on laboratory scale.

The velocity and temperature profiles for one-dimensional flow are

1 $$u=[0,0,w(r)],{\bf{S}}={\bf{S}}(r),{\rm{\Theta }}={\rm{\Theta }}(r).$$

Here *R*_*d*_ is the radius of the die, ${\theta }_{w}$ temperature of the wire and ${\theta }_{d}$ is the temperature of the die.

For viscoelastic fluid, the stress tensor is:

2 $$\begin{array}{c}{\bf{S}}+{\gamma }_{1}\frac{D{\bf{S}}}{Dt}+\frac{{\gamma }_{3}}{2}({{\bf{A}}}_{1}{\bf{S}}+{\bf{S}}{{\bf{A}}}_{1})+\frac{{\gamma }_{5}}{2}(tr{\bf{S}}){{\bf{A}}}_{1}+\frac{{\gamma }_{6}}{2}(tr{\bf{S}}{{\bf{A}}}_{1}){\bf{I}}\\ \,=\,\eta ({{\bf{A}}}_{1}+{\gamma }_{2}\frac{D{{\bf{A}}}_{1}}{Dt}+{\gamma }_{4}{{\bf{A}}}_{1}^{2}+\frac{{\gamma }_{7}}{2}(tr{{\bf{A}}}_{1}^{2}){\bf{I}}),\end{array}$$

In above equation ${{\bf{A}}}_{1}$ and ${\gamma }_{i}(i=1\,-\,7)$ are the Rivlin-Ericksen tensor and material constants respectively.

3 $${{\rm{A}}}_{1}={L}^{T}+L,$$

4 $${{\rm{A}}}_{n}={{\rm{A}}}_{n-1}{L}^{T}+L{{\rm{A}}}_{n-1}+\frac{D{{\rm{A}}}_{n-1}}{Dt},n=2,3,\mathrm{...}$$

The basic governing equations for incompressible flow are the continuity, momentum and energy equations are given by:

5 $$\nabla .u=0,$$

6 $$\rho \frac{Du}{Dt}=\nabla .{\rm{T}}+{\rm{J}}\times {\rm{B}},$$

7 $$\begin{array}{c}\rho {c}_{p}\frac{D\theta }{Dt}=k{\nabla }^{2}\theta +{\rm{T}}.{\rm{L}},\end{array}$$

In Eq. (6) the body force is defined as.

8 $$J\times B=-\,\sigma {B}_{0}^{2}w.$$

In view of Eqs (1–8), we have:

9 $$\frac{\partial p}{\partial z}=\frac{1}{r}\frac{d}{dr}(r{S}_{rz}),$$

10 $$\frac{\partial p}{\partial \theta }=0,$$

11 $$\frac{\partial p}{\partial z}=\frac{1}{r}\frac{d}{dr}(r{S}_{rz})-\sigma {B}_{0}^{2}w-\frac{\nu }{{k}_{p}}w,$$

12 $$\frac{\partial p}{\partial z}=\frac{1}{r}\frac{d}{dr}[\frac{r\eta (1+\alpha {(\frac{dw}{dr})}^{2})}{(1+\beta {(\frac{dw}{dr})}^{2})}],$$

13 $$k(\frac{{d}^{2}\theta }{d{r}^{2}}+\frac{1}{r}\frac{d\theta }{dr})[\frac{r\eta (1+\alpha {(\frac{dw}{dr})}^{2})}{(1+\beta {(\frac{dw}{dr})}^{2})}]=0,$$

where

$$\alpha ={\gamma }_{1}({\gamma }_{4}+{\gamma }_{7})-({\gamma }_{3}+{\gamma }_{5})({\gamma }_{4}+{\gamma }_{7}-{\gamma }_{2})-\frac{{\gamma }_{5}{\gamma }_{7}}{2},$$

$$\beta ={\gamma }_{1}({\gamma }_{3}+{\gamma }_{6})-({\gamma }_{3}+{\gamma }_{5}){\gamma }_{1}({\gamma }_{3}+{\gamma }_{6}-{\gamma }_{1})-\frac{{\gamma }_{5}{\gamma }_{6}}{2}.$$

### Constant viscosity

Introducing the dimensionless parameters

14 $$\begin{array}{c}{r}^{\ast }=\frac{r}{{R}_{w}},{w}^{\ast }=\frac{w}{V},{\alpha }^{\ast }=\frac{\alpha {V}^{2}}{{R}_{w}^{2}},{\beta }^{\ast }=\frac{\beta {V}^{2}}{{R}_{w}^{2}},M=\frac{\sigma {B}_{0}^{2}}{(\eta /{R}_{w}^{2})},\\ {p}^{\ast }=\frac{p}{(\eta V/{R}_{w}^{2})},{\beta }_{r}=\frac{{\mu }_{0}{U}_{w}^{2}}{k({\theta }_{d}-{\theta }_{w})},{k}_{p}^{\ast }=\frac{{k}_{p}}{{R}_{w}^{2}},\\ {\theta }^{\ast }=\frac{\theta -{\theta }_{w}}{{\theta }_{d}-{\theta }_{w}},Br=\frac{\eta {V}^{2}}{k({\theta }_{d}-{\theta }_{w})}.\end{array}$$

In view of Eq. (14), the system of Eqs (12) and (13) becomes:

15 $$\begin{array}{rcl}r{\rm{\Omega }} & = & \tfrac{[\tfrac{dw}{dr}+(\alpha +\beta ){(\tfrac{dw}{dr})}^{3}+\alpha \beta {(\tfrac{dw}{dr})}^{5}]+r[\tfrac{{d}^{2}w}{d{r}^{2}}+(3\alpha -\beta ){(\tfrac{dw}{dr})}^{2}(\tfrac{{d}^{2}w}{d{r}^{2}})+\alpha \beta {(\tfrac{dw}{dr})}^{4}\tfrac{{d}^{2}w}{d{r}^{2}}]}{{[1+\beta {(\tfrac{dw}{dr})}^{2}]}^{3}}\\  &  & +\frac{1}{\mu }\frac{d\mu }{dr}r[\frac{\frac{dw}{dr}+\alpha {(\frac{dw}{dr})}^{3}}{1+\beta {(\frac{dw}{dr})}^{2}}]-\frac{1}{\mu }[Mwr+\frac{1}{Kp}wr],\end{array}$$

16 $$[\frac{{d}^{2}\theta }{d{r}^{2}}+\frac{1}{r}\frac{d\theta }{dr}]+\frac{\mu {\beta }_{r}{(\frac{dw}{dr})}^{2}[[1+\beta {(\frac{dw}{dr})}^{2}]]}{[1+\beta {(\frac{dw}{dr})}^{2}]}=0$$

Corresponding to the boundary-conditions

17 $$w(1)=1,w(\delta )=0,$$

18 $$\theta (1)=0,\theta (\delta )=1.$$

The constant viscosity case is discussed by Shah *et al*.^46^ in detail. Here we discuss the variable

viscosity case.

### Reynolds model

In this case $\eta$ is a function of temperature. Introducing the dimensionless parameters:

19 $$\begin{array}{c}{r}^{\ast }=\frac{r}{{R}_{w}},{w}^{\ast }=\frac{w}{V},{\alpha }^{\ast }=\frac{\alpha {V}^{2}}{{R}_{w}^{2}},\frac{{R}_{d}}{{R}_{w}}=\delta  > 1,{\beta }^{\ast }=\frac{\beta {V}^{2}}{{R}_{w}^{2}},\\ M=\frac{\sigma {B}_{0}^{2}}{(\eta /{R}_{w}^{2})},{p}^{\ast }=\frac{p{R}_{w}}{(\eta V)},Br=\frac{{\mu }_{0}{U}_{w}^{2}}{k({\theta }_{d}-{\theta }_{w})},{k}_{p}^{\ast }=\frac{{k}_{p}}{{R}_{w}^{2}},\\ {\theta }^{\ast }=\frac{\theta -{\theta }_{w}}{{\theta }_{d}-{\theta }_{w}},Br=\frac{\eta {V}^{2}}{k({\theta }_{d}-{\theta }_{w})},\frac{\partial p}{\partial z}={\rm{\Omega }}.\end{array}$$

In Reynolds model, the dimensionless temperature dependent viscosity can be expressed as $\eta ={e}^{(-{B}_{0}m\theta )}$ $\approx 1-{B}_{0}m\theta$. Therefore, Eqs (15–18) in view of Eq. (19) becomes:

20 $$\begin{array}{rcl}\frac{{d}^{2}w}{d{r}^{2}} & = & \tfrac{r(1+\beta {(\tfrac{dw}{dr})}^{2})[{\rm{\Omega }}+\tfrac{1}{(1-{\beta }_{0}m\theta )}(M+\tfrac{1}{Kp})w+\tfrac{1}{(1-{\beta }_{0}m\theta )}{\beta }_{0}m(\tfrac{d\theta }{dr})(\tfrac{\tfrac{dw}{dr}+\alpha {(\tfrac{dw}{dr})}^{3}}{(1+\beta {(\tfrac{dw}{dr})}^{2})})]}{r[1+(3\alpha -\beta ){(\tfrac{dw}{dr})}^{2}+\alpha \beta {(\tfrac{dw}{dr})}^{4}]}\\  &  & -\frac{[\frac{dw}{dr}+(\alpha +\beta ){(\frac{dw}{dr})}^{3}+\alpha \beta {(\frac{dw}{dr})}^{5}]}{r[1+(3\alpha -\beta ){(\frac{dw}{dr})}^{2}+\alpha \beta {(\frac{dw}{dr})}^{4}]},\end{array}$$

21 $$\frac{{d}^{2}\theta }{d{r}^{2}}=-\,\frac{1}{r}\frac{d\theta }{dr}-(1-{B}_{0}m\theta )\frac{{B}_{r}({(\frac{dw}{dr})}^{2}+\alpha {(\frac{dw}{dr})}^{4})}{(1+\beta {(\frac{dw}{dr})}^{2})},$$

22 $$\begin{array}{c}w(1)=1,w(\delta )=0,\\ \theta (0)=0,\theta (\delta )=1.\end{array}$$

### Vogel’s model

In this case of temperature dependent viscosity, temperature can be expressed as $\mu ={\mu }_{0}\exp (\frac{D}{{B}^{\text{'}}+\theta }-{\theta }_{w})$, by expanding

23 $$\eta ={{\rm{\Omega }}}_{1}(1-\frac{D}{{B}_{1}^{2}}\theta ),$$

where ${{\rm{\Omega }}}_{1}={\eta }_{0}\exp (\frac{D}{{B}^{\text{'}}}-{\theta }_{w})$ and $D,$ $B^{\prime}$ denotes the temperature dependent viscosity parameters of the Vogel’s model. Therefore, Eqs (15–18) in view of Eq. (19) becomes:

24 $$\begin{array}{rcl}\frac{{d}^{2}w}{d{r}^{2}} & = & \tfrac{r(1+\beta {(\tfrac{dw}{dr})}^{2})[{\rm{\Omega }}+\tfrac{1}{{{\rm{\Omega }}}_{1}(1-\tfrac{D}{{B}_{1}^{2}}\theta )}(M+\tfrac{1}{Kp})w+\tfrac{D}{{B}_{1}^{2}}(\tfrac{d\theta }{dr})(\tfrac{\tfrac{dw}{dr}+\alpha {(\tfrac{dw}{dr})}^{3}}{(1+\beta {(\tfrac{dw}{dr})}^{2})})]}{r[1+(3\alpha -\beta ){(\tfrac{dw}{dr})}^{2}+\alpha \beta {(\tfrac{dw}{dr})}^{4}]}\\  &  & -\frac{[\frac{dw}{dr}+(\alpha +\beta ){(\frac{dw}{dr})}^{3}+\alpha \beta {(\frac{dw}{dr})}^{5}]}{r[1+(3\alpha -\beta ){(\frac{dw}{dr})}^{2}+\alpha \beta {(\frac{dw}{dr})}^{4}]},\end{array}$$

25 $$\frac{{d}^{2}\theta }{d{r}^{2}}=-\,\frac{1}{r}\frac{d\theta }{dr}-{{\rm{\Omega }}}_{1}(1-\frac{D}{{B}_{1}^{2}}\theta )\frac{{B}_{r}({(\frac{dw}{dr})}^{2}+\alpha {(\frac{dw}{dr})}^{4})}{(1+\beta {(\frac{dw}{dr})}^{2})},$$

26 $$\begin{array}{c}w(1)=1,w(\delta )=0,\\ \theta (0)=0,\theta (\delta )=1.\end{array}$$

## Numerical Solution

The above system of equations are solved numerically by using fourth order Runge-Kutta method along with shooting technique. For this purpose the Eqs (20), (21), (24) and (26) are transformed in to first order due to the higher order equation at $r=\delta$ (boundary-layer thickness) unavailable. Then, the boundary value problem is solved by shooting method.

### Numerical solution of Reynolds model

Eqs (20) and (21) are solved numerically by Ruge-Kutta method corresponding to the boundary conditions (22) by considering

27 $${z}_{1}=w,{z}_{2}=\frac{dw}{dr}\,{\rm{and}}\,{{\rm{z}}}_{3}=\theta ,{z}_{4}=\frac{d\theta }{dr}.$$

In view of Eq. (27), Eqs (20) and (22) becomes:

28 $$\begin{array}{c}{z^{\prime} }_{2}=\tfrac{[r({\rm{\Omega }}+\tfrac{1}{(1-{\beta }_{0}m\theta )}(M+\tfrac{1}{Kp})){z}_{1}+\tfrac{1}{(1-{\beta }_{0}m\theta )}{\beta }_{0}m{z}_{4}(\tfrac{{z}_{2}+\alpha {z}_{2}^{3}}{(1+\beta {z}_{2}^{2})})]{(1+\beta {z}_{2}^{2})}^{2}-[{z}_{2}+(\alpha +\beta ){z}_{2}^{3}+\alpha \beta {z}_{2}^{5}]}{r[1+(3\alpha -\beta ){z}_{2}^{2}+\alpha \beta {z}_{2}^{4}]}\\ {z^{\prime} }_{4}=-\frac{1}{r}{z}_{4}-(1-{B}_{0}m\theta )\frac{{B}_{r}({z}_{2}^{2}+\alpha {z}_{2}^{4})}{(1+\beta {z}_{2}^{2})},\end{array}$$

with boundary conditions

29 $$\begin{array}{c}{z}_{a}(1)=1,{z}_{b}(1)=0,\\ {z}_{a}(2)=0,{z}_{b}(2)=1.\end{array}$$

### Numerical solution of Vogel’s Model

The numerical solution of Eqs (24) and (25) with boundary conditions given in Eq (26) can be obtained by considering $D={\beta }_{0}b$ The expression for the velocity and temperature profiles are:

30 $${z^{\prime} }_{2}=\tfrac{[r({\rm{\Omega }}+\tfrac{1}{{{\rm{\Omega }}}_{1}(1-\tfrac{D}{{B}_{1}^{2}}\theta )}(M+\tfrac{1}{Kp})){z}_{1}+\tfrac{\tfrac{D}{{B}_{1}^{2}}{z}_{4}}{{{\rm{\Omega }}}_{1}(1-\tfrac{D}{{B}_{1}^{2}}\theta )}(\tfrac{{z}_{2}+\alpha {z}_{2}^{3}}{(1+\beta {z}_{2}^{2})})]{(1+\beta {z}_{2}^{2})}^{2}-[{z}_{2}+(\alpha +\beta ){z}_{2}^{3}+\alpha \beta {z}_{2}^{5}]}{r[1+(3\alpha -\beta ){z}_{2}^{2}+\alpha \beta {z}_{2}^{4}]}$$

31 $${z^{\prime} }_{4}=-\frac{1}{r}{z}_{4}-{{\rm{\Omega }}}_{1}(1-\frac{D}{{B}_{1}^{2}}\theta )\frac{{B}_{r}({z}_{2}^{2}+\alpha {z}_{2}^{4})}{(1+\beta {z}_{2}^{2})},$$

with the specific boundary conditions

32 $$\begin{array}{c}{z}_{a}(1)=1,{z}_{b}(1)=0,\\ {z}_{a}(2)=0,{z}_{b}(2)=1.\end{array}$$

## Analysis of the Results

In this section the results are discussed derived on the theoretical basis. In the following subsection the figures illustrate how the fluid velocity and the temperature distribution vary in the pressure unit of the conical geometry for different physical parameter values such as dilatant constant $(\alpha ),$ pseudoplastic constant $(\beta ),$ viscosity parameter of Reynolds model $(m),$ and Vogel’s parameter $({\rm{\Omega }}),$ Brinkman number $(Br),$ magnetic parameter $(M)$ and porosity parameter $({k}_{p}).$ The numerical solutions have been calcultaed by fourth order Ruge-Kutta method along with shooting technique.

### Reynolds Model

**Figure 2:**
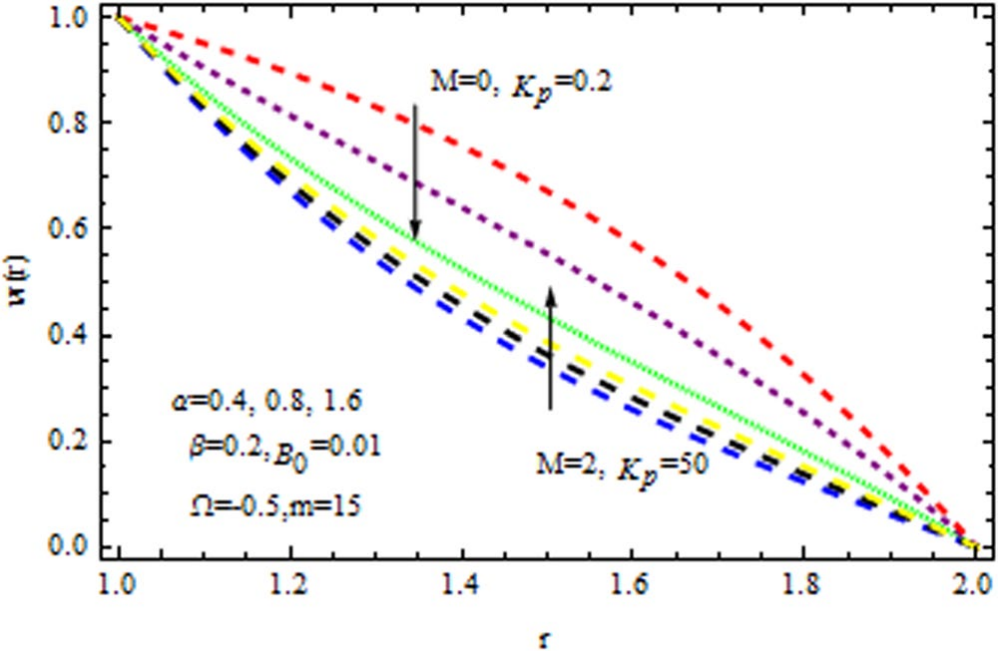
Impact of $\alpha$ on velocity (Reynolds model).

**Figure 3:**
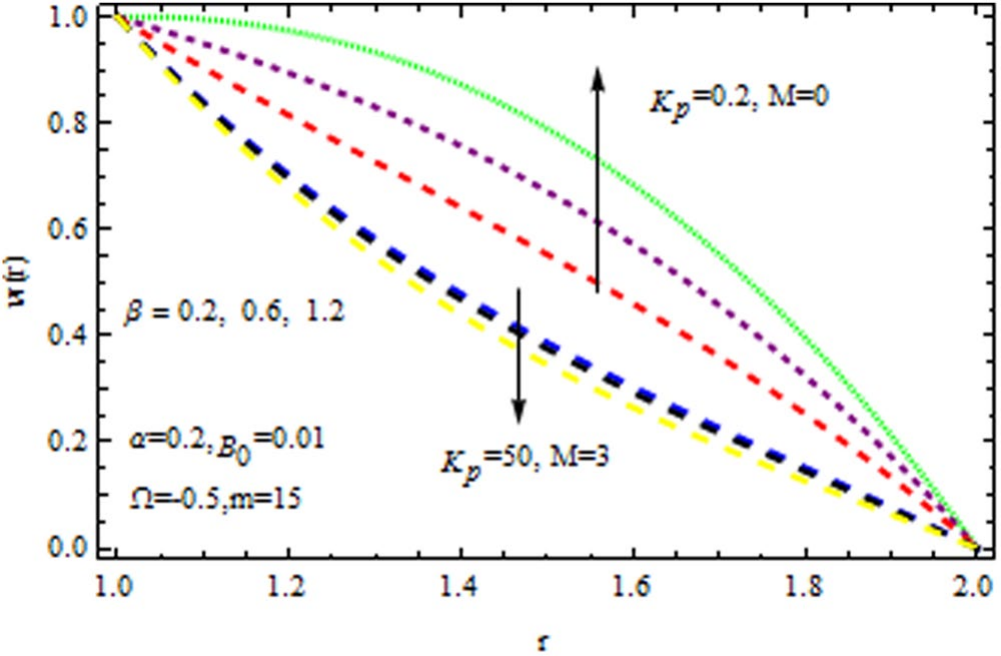
Impact of $\beta$ on velocity (Reynolds model).

Figure 2 displays the velocity profile in he presence and absence of porous matrix $({k}_{p}).$ by variation of magnetic parameter $(M)$ and dilatant constant $(\alpha ).$ The effect of dilatant constant on the velocity profile is significant. It is noticed that the velocity profile retards with the increasing values of dilatant constant but reverse effect is observed in the presence of ${k}_{p}\,{\rm{and}}\,M=0.$ For $M=2,$ the situation becomes adverse. It is interesting to note that for α = 0.4 the present result made significant agreement with the reported results^45^ when $M=0,\,{{\rm{k}}}_{p}=\mathrm{0.2.}$ The impact of $\beta$ on velocity varaition is depicted in Fig. 3 in the presence and absence of $M\,{\rm{and}}\,{k}_{p}.$ It is investigated that the pseudoplastic constant $(\beta ),$ accelerates the velocity of the coating polymer by taking $M=0.$ But converse effect is detected in the presence of ${{\rm{k}}}_{p}$. Thus in the absence of $M\,{\rm{and}}\,{{\rm{k}}}_{p}$ the non-Newtonian parameter accelerates the velocity profile within the die. It is also evident that for $M=3,$ the velocity profile retards as $\beta$ increases in the presence/absence of ${{\rm{k}}}_{p}$.

**Figure 4:**
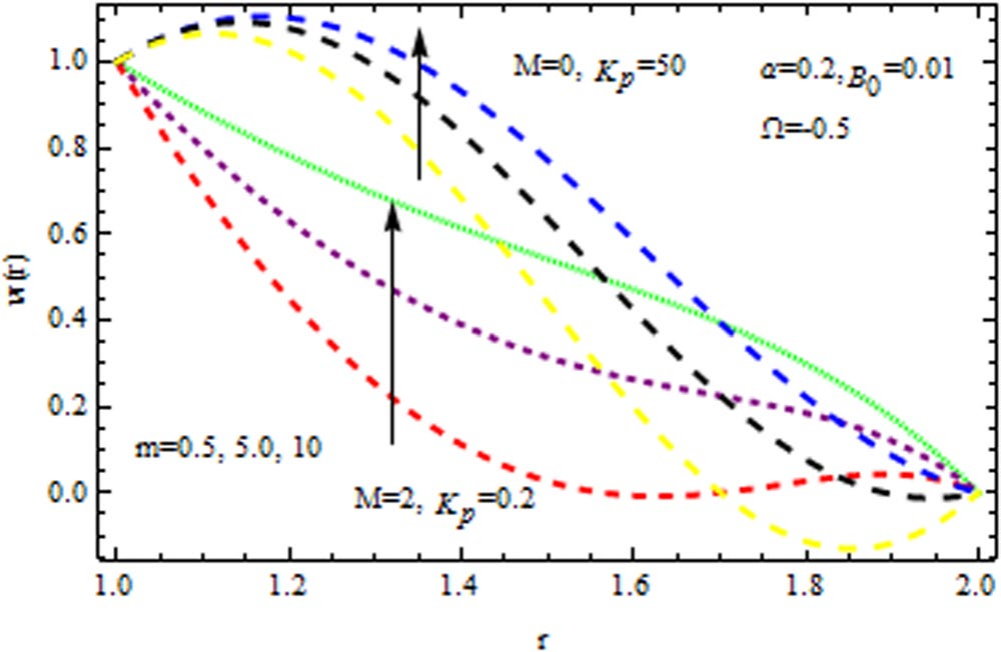
Impact of $m$ on velocity (Reynolds model).

**Figure 5:**
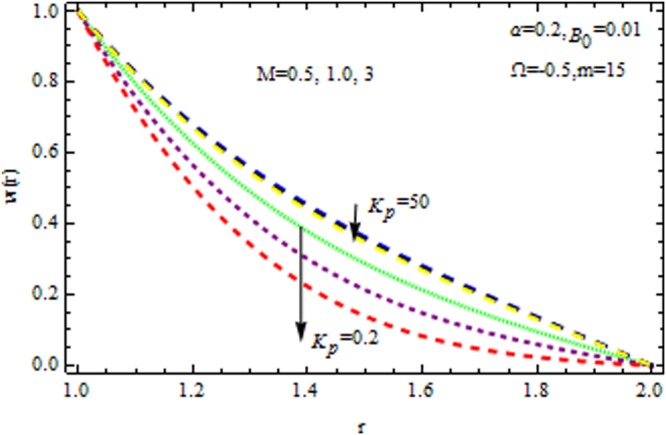
Impact of $M$ on velocity (Reynolds model).

Figure 4 displays the influence of viscosity parameter (Reynolds model parameter) $m$ on the velocity profile in the presence/absence of ${{\rm{k}}}_{p}$. It is observed that for both presence/absence of ${k}_{p}\,{\rm{and}}\,M$ the velocity profile increases all points. Due to high magnetic field, the electromagnetic force may add to nonlinearity of velocity distribution. It is also stimulated that for high values of viscosity and magnetic parameter i.e., ${k}_{p}=\mathrm{50}\,{\rm{and}}\,M=2,$ accelerates/decelerates the velocity profile. Figure 5 delineates the impact of $M$ on velocity profile in the presence/absence of ${{\rm{k}}}_{p}$. It is perceived that the velocity profile decreases with magnetic parameter. This deceleration in velocity field is due to the magnetic force density which is equivalent to a viscous force means orthogonal to the direction of magnetic field.

**Figure 6:**
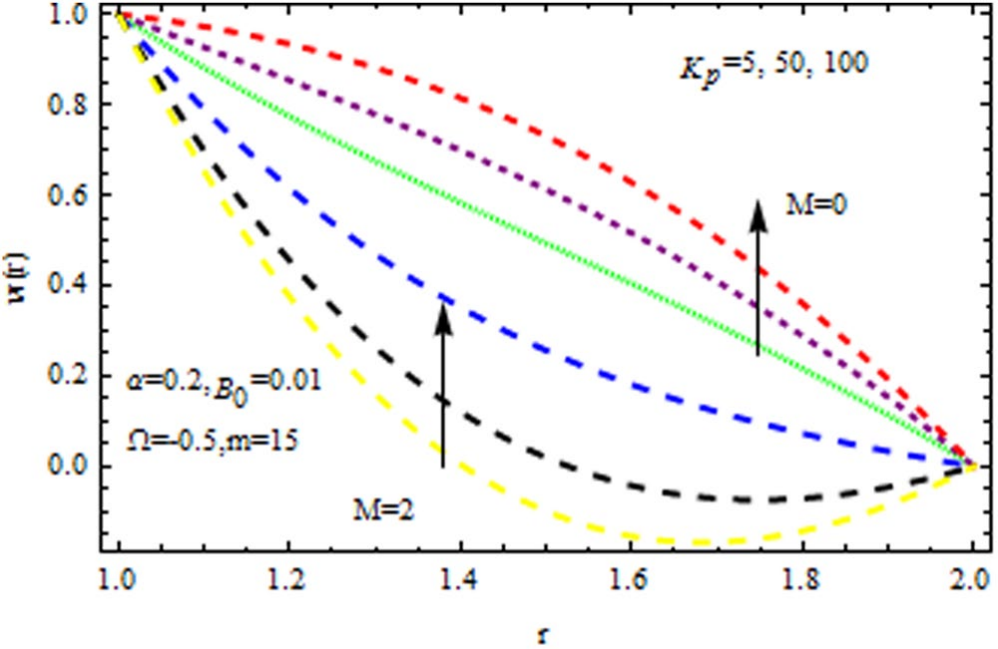
Impact of ${k}_{p}$ on velocity (Reynolds model).

**Figure 7:**
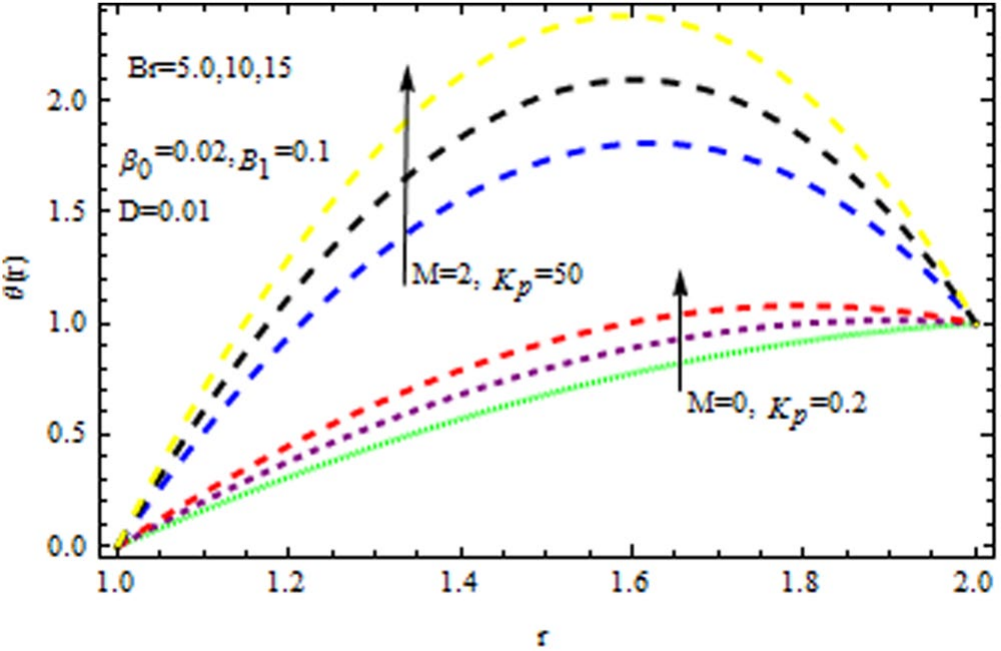
Impact of $Br$ on temperature (Reynolds model).

**Figure 8:**
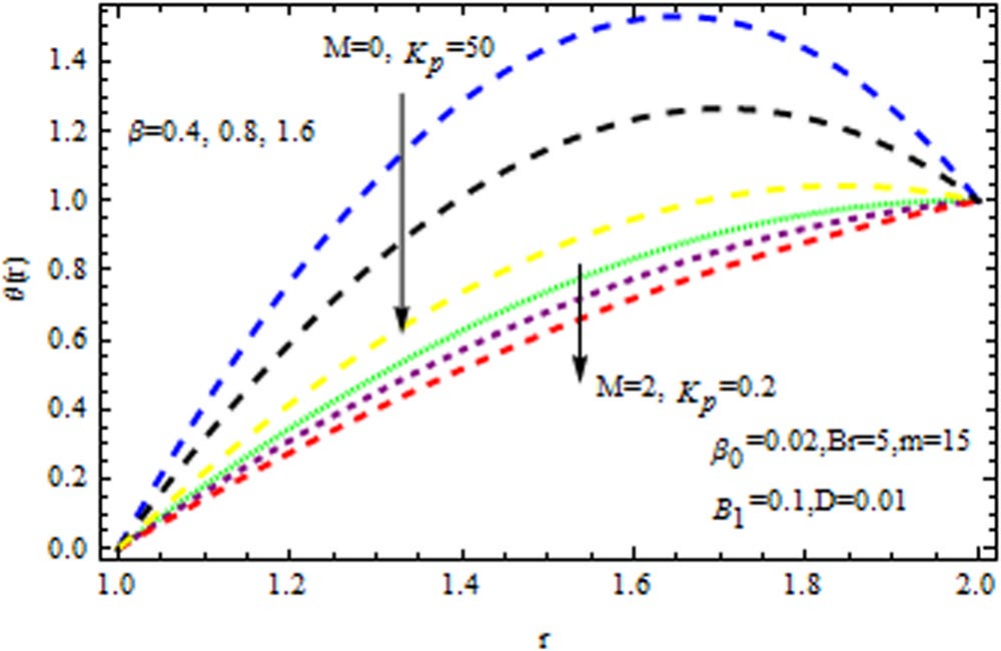
Impact of $\beta$ on temperature (Reynolds model).

**Figure 9:**
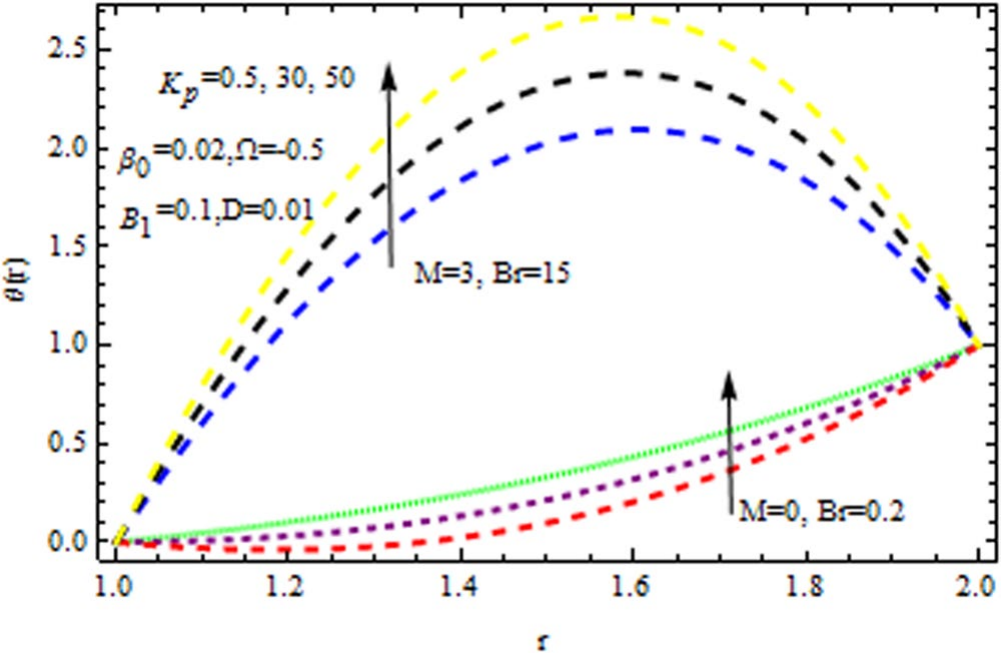
Impact of ${k}_{p}$ on temperature (Reynolds model).

In the presence and absence of $M,$ Fig. 6 depicts the velocity profile for various values of porous matrix. This shows that the porous matrix has accelerating effect of velocity profile both in the presence and absence of $M.$ The impact of $Br$ and $M$ in the presence/absence of porous medium on the temperature profile is revealed in Fig. 7. It follows that the $Br$ i.e., the relation between viscous heating to the heat conductor enhance the temperature distribution in the presence/absence of ${{\rm{k}}}_{p}$. It is also remarkable to note that two-layer variation is noted with the increasing values of magnetic parameter. Temperature distribution increases sharply within the region $r\le 1.4,$ and retards significantly. In the absence/presence of ${{\rm{k}}}_{p}$, the impact of $M$ and $\beta$ on the temperature distribution is displayed in Fig. 8. The Reynolds model viscosity parameter is taken to be fixed at $m=15.$ It is seen that the temperature decreases with increasing $\beta$ in the presence/absence of ${k}_{p}\,{\rm{and}}\,M$. Figure 9 sows the effect of porous matrix on temperature distribution. It is noticed that the temperature profile gets accelerated as ${k}_{p}$ increase in both ceases. Also is remarkable to note that for large values of magnetic parameter and Brinkman number i.e., $M=3\,{\rm{and}}\,Br=15,$ the temperature is maximum at the middle of the annular die.

### Vogel’s model

**Figure 10:**
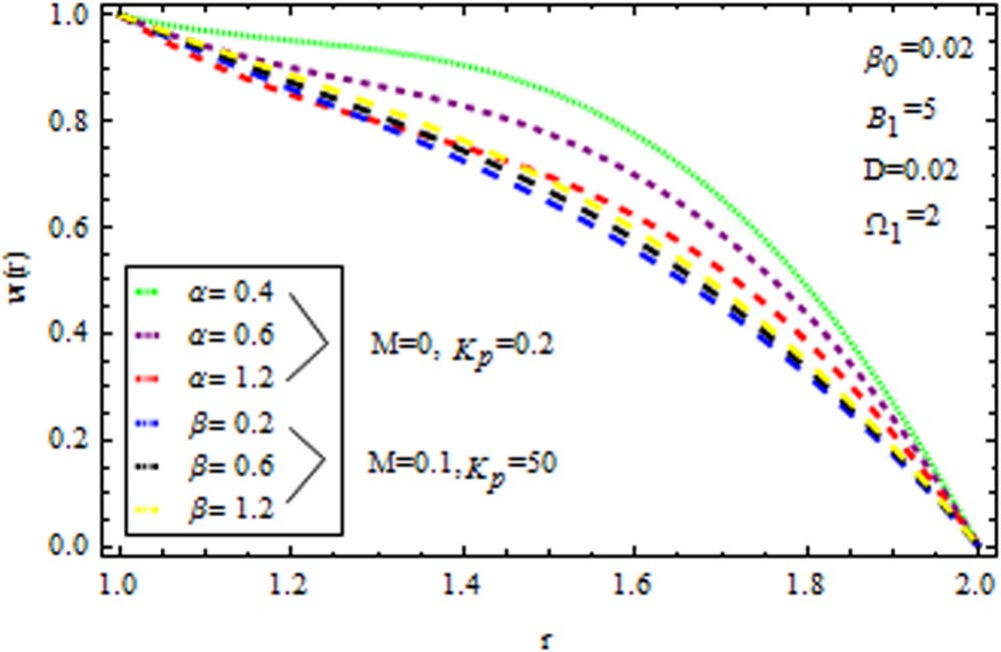
Impact of $\alpha \,{\rm{and}}\,\beta$ on velocity (Vogel’s model).

**Figure 11:**
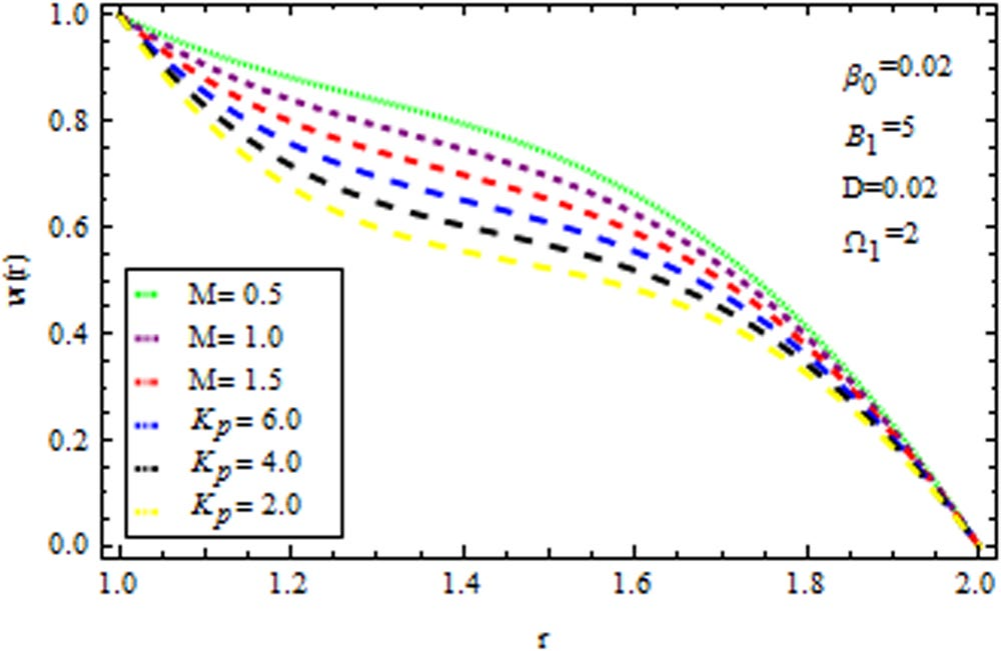
Impact of $M\,{\rm{and}}\,{{\rm{k}}}_{p}$ on velocity (Vogel’s model).

**Figure 12:**
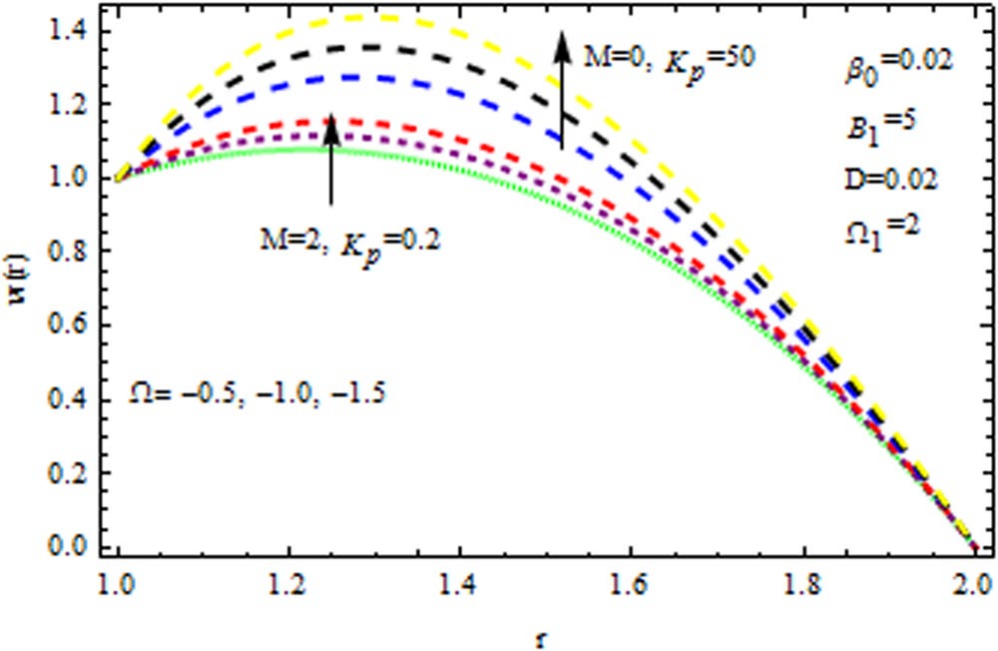
Impact of Ω on velocity profile (Vogel’s model).

**Figure 13:**
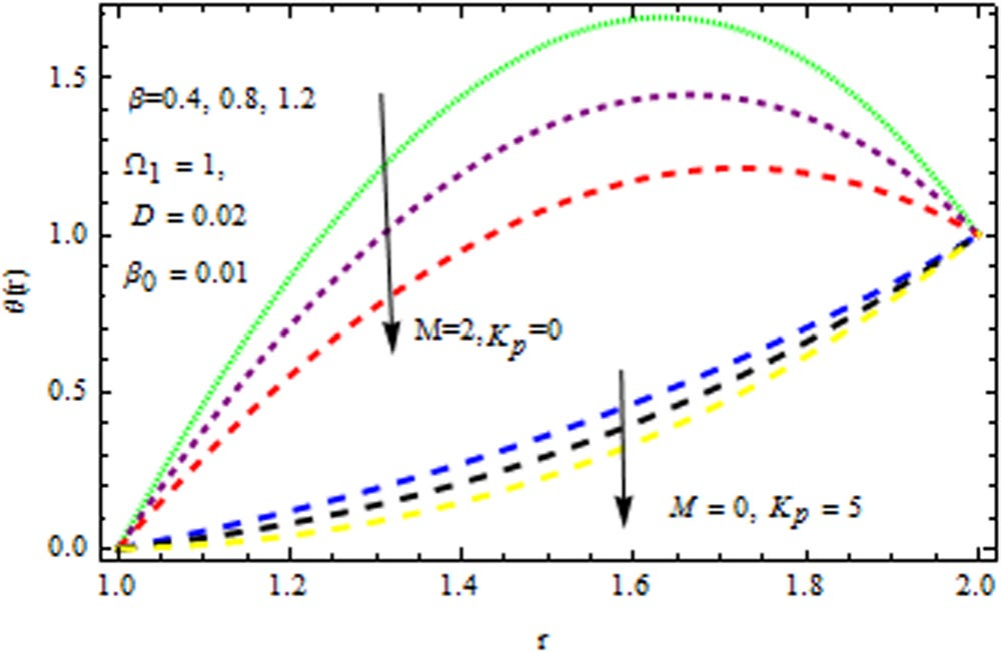
Impact of *β* on temerature (Vogel’s model).

**Figure 14:**
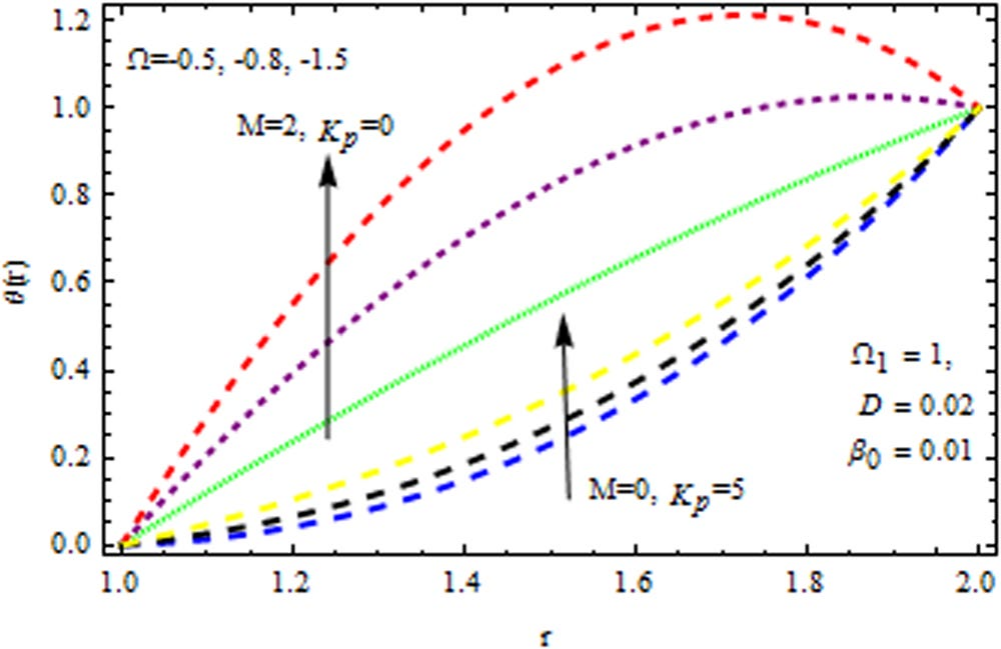
Impact of Ω on temerature (Vogel’s model).

**Figure 15:**
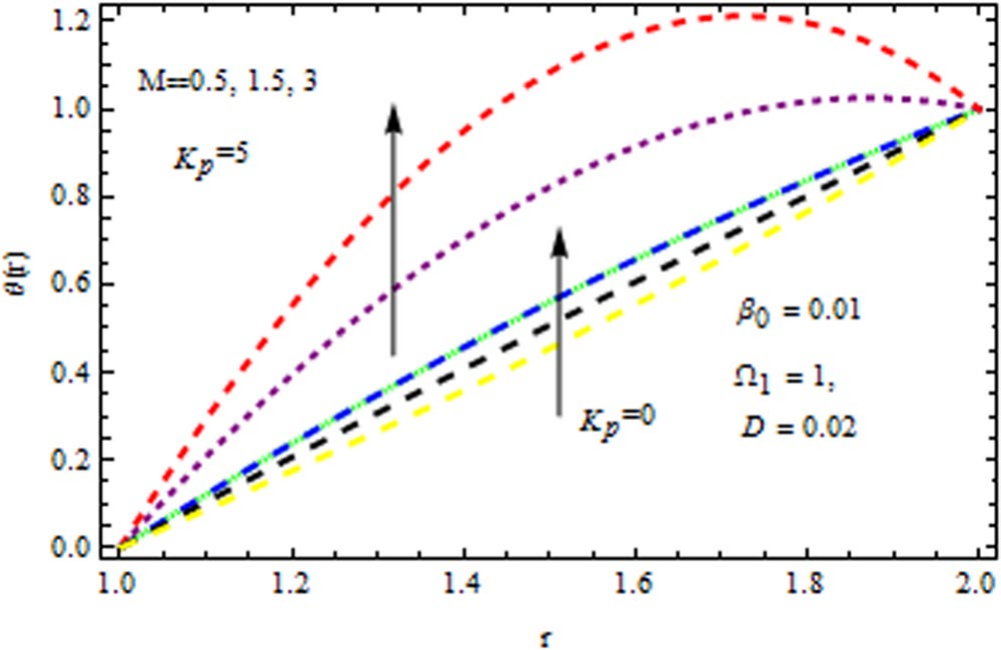
Impact of *M* on temerature (Vogel’s model).

**Figure 16:**
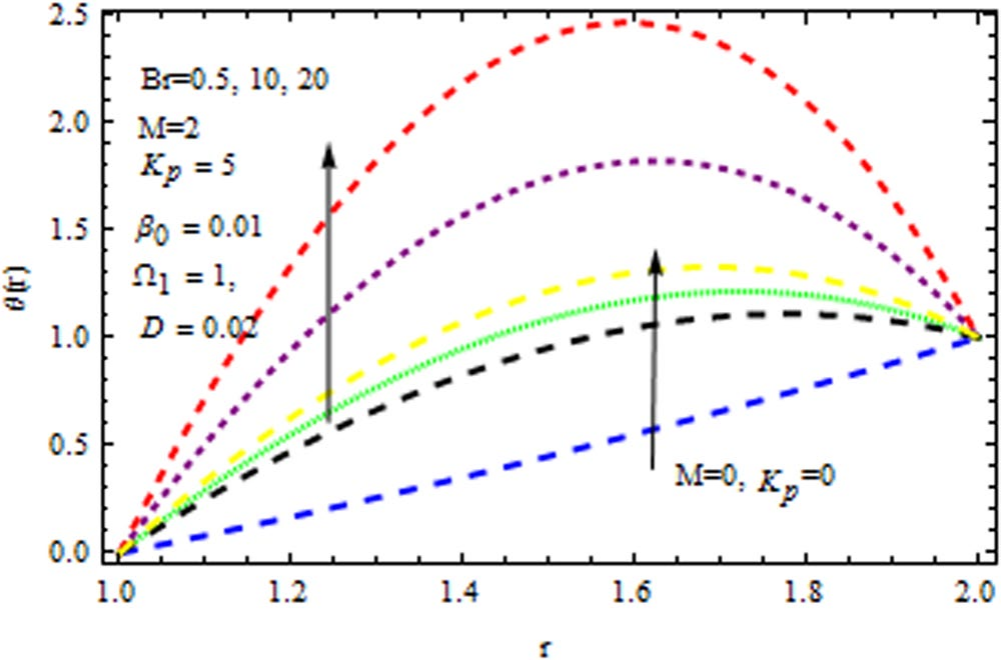
Impact of *Br* on temerature (Vogel’s model).

**Figure 17:**
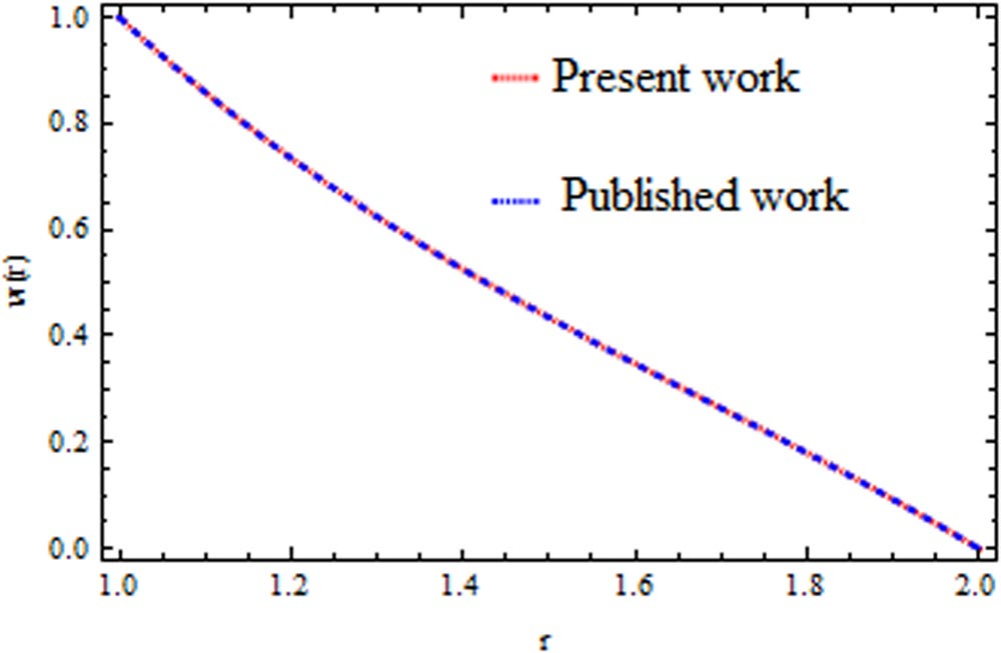
Velocity comparison of the present work with published work^45^.

The effect of physical parameter involves in the Vogel’s model on the solutions are revealed in Figs 10–16. The Fig. 10 shows the effect of dilatant constant $\alpha$ as well as the pseudoplastic constant $\beta$ in the presence/absence of ${k}_{p}\,{\rm{and}}\,M$ on the velocity. From this simulation it’s pointed out that the velocity decreases as the dilatant constant increases while the effect of $\beta$ is quite opposite to that of $\alpha$. Also the results of Shah *et al*.^45^ can be easily recovered by taking $M=0,{k}_{p}=0,\alpha =\mathrm{0.4.}$ The variation of the velocity of the coating liquid is displayed in Fig. 11. It is seen that the velocity decreases with increasing $M$. This due the Lorenz forces, which act as a resistive force and resist the motion of theffluid. The variation of the velocity is significant for porous matrix. Figure 12 depicts the effect of $M$ and ${\rm{\Omega }}$ (pressure gradient parameter of Vegel’s model) on the velocity profile in the presence and absence of ${{\rm{k}}}_{p}$. Due the existence of non-Newtonian characteristic the pressure gradient parameter accelerates the velocity profile significantly in the domain $r\le 1.3$ and then decrease sharply. In the presence/absence of porous matrix as well as magnetic parameter, the effect of pseudoplastic constant on temperature is displayed in Fig. 13. With the increasing values of $\beta$ the temperature distribution within the die increases. It is interesting to note that for $M=3$ and ${{\rm{k}}}_{p}=0.2$, the temperature profiles increases significantly to a certain region $r\le 1.5$ and afterwards, it decreases. Figure 14 reveals the effect of pressure gradient parameter of Vogel’s model on the temperature profile with the contribution of constant porous matrix in the presence/absence of $M$. From this simulation it is observed that $M=0\,{\rm{and}}\,{{\rm{k}}}_{p}=0.2,$ the temperature profile increases as Ω increases. Retarding effect was observed after the region $r\ge 1.5$ and in the presence of $M$ and ${{\rm{k}}}_{p}$ reverse effect was encountered near the plate. An interesting observation is observed in Fig. 15. It is significant that the temperature distribution gets accelerated due to the increase of $M$ in the presence and absence of ${{\rm{k}}}_{p}$. The impact of $Br$ on the temperature is depicted in Fig. 16. It is observed that for large value of Brinkman number i.e., Br = 20, the peak in temperature distribution is encountered within the layer $1\le r\le 1.7$ and afterwards, it decreased sharply in the presence and absence of ${{\rm{k}}}_{p}$. Finally, the present results are also compared with reported results^45^ and good agreement has been found as presented in Table 1 and in Fig. 17.

**Table 1:** Comparison of present work with Rehan *et al*.^45^.

r	Present work	Rehan *et al*.^45^
1	1	1
1.2	0.734425	0.73451
1.4	0.525222	0.525203
1.6	0.348501	0.348552
1.8	0.180476	0.180466
2	0	0

## Conclusion

The magnetic field and heat transfer effect on wire coating analysis is investigated numerically while with drawing the wire from the center of he die filled with viscoelastic fluid. The velocity and temperature profiles have been obtained numerically by applying Ruge-Kutta method along with shooting technique. The effect of variable viscosity is also encountered. Reynolds and Vogel’s models are used for the variable viscoasity. The effect of physical parameters such as values such as dilatant constant $(\alpha ),$ pseudoplastic constant $(\beta ),$ viscosity parameter of Reynolds model $(m),$ and viscosity parameter of Vogel’s model $({\rm{\Omega }})$, Brinkman number $(Br),$ magneticpparameter $(M)$ and porositypparameter $({k}_{p}).$ It is seen that the non-Newtonian parameter of the fluid accelerates the velcoty profile in the absence of porous and magnetic parameters. For large value of magnetic parameter the reverse effect is observed. It is observed that the temperature profiles decreases with increasing psedoplastic parameter in the presence and absence of porous matrix as well as magnetic parameter. The Brinkman number contributes to increase the temperature for both Reynolds and Vogel’smmodels. With the increasing of pressure gradient parameter of both Reynolds and Vogel’s models, the velocity and temperature profile increases significantly in the presence of non-Newtonian parameter.
